# Microbially driven organic carbon cycling at the land−sea interface: Advances and an integrated study framework

**DOI:** 10.1002/mlf2.70082

**Published:** 2026-05-18

**Authors:** Quanrui Chen, Kai Tang, Zhili He, Meng Li, Jun Yang, Weidong Zhai, Qiang Zheng, Nianzhi Jiao

**Affiliations:** ^1^ Innovation Research Center for Carbon Neutralization, State Key Laboratory of Marine Environmental Science, Fujian Key Laboratory of Marine Carbon Sequestration, College of Ocean and Earth Sciences Xiamen University Xiamen China; ^2^ Frontier Research Center, Southern Marine Science and Engineering Guangdong Laboratory (Zhuhai) Zhuhai China; ^3^ Archaeal Biology Center, Synthetic Biology Research Center, Shenzhen Key Laboratory of Marine Microbiome Engineering, Key Laboratory of Marine Microbiome Engineering of Guangdong Higher Education Institutes, Institute for Advanced Study Shenzhen University Shenzhen China; ^4^ Aquatic EcoHealth Group, Fujian Key Laboratory of Watershed Ecology, State Key Laboratory of Regional and Urban Ecology, Institute of Urban Environment Chinese Academy of Sciences Xiamen China

**Keywords:** carbon cycle, dissolved organic carbon, land−sea interface, microbial carbon pump, microbial processes

## Abstract

The land−sea interface is a vital component of global biogeochemical cycles, where microorganisms drive the cycling of carbon, nitrogen, and sulfur. This review synthesizes the research progress from representative land−sea interfaces to elucidate how the microbial community structure and metabolic function influence the mobilization, transformation, and retention of organic carbon. Here, we also review the mechanisms underlying carbon cycle dynamics and emphasize the role of coupled biogeochemical cycles and climate change. A key focus is the synergistic interaction among the marine microbial carbon pump (MCP), the soil MCP, and the mineral‐associated carbon pump, hereafter referred to as the land−sea MCP framework. We further propose an integrated study framework, based on measurable parameters such as carbon use efficiency and bacterial growth efficiency, to link microbial processes to long‐term carbon sequestration at the land−sea interface.

## INTRODUCTION

The land−sea interface, encompassing estuaries, coastal wetlands, intertidal zones, and adjacent continental seas^1^, ^2^, forms a critical junction between terrestrial and marine carbon pools, receiving significant fluxes of dissolved organic carbon (DOC) and particulate organic carbon (POC) from riverine and wetland systems^3^, ^4^, ^5^, ^6^. Inland freshwater systems, including rivers, lakes, and reservoirs, are considered upstream processors because they regulate the quantity, lability, and partitioning of carbon delivered to the coastal interface. Neglecting carbon dynamics in this zone may lead to a significant overestimation of terrestrial carbon sequestration and an underestimation of marine carbon storage^1^. Moreover, carbon cycling in these regions is increasingly disrupted by human activities, resulting in environmental stressors, such as eutrophication, hypoxia, and acidification, yielding a paradox of high carbon fixation but low carbon sequestration^7^, ^8^.

**Figure 1:**
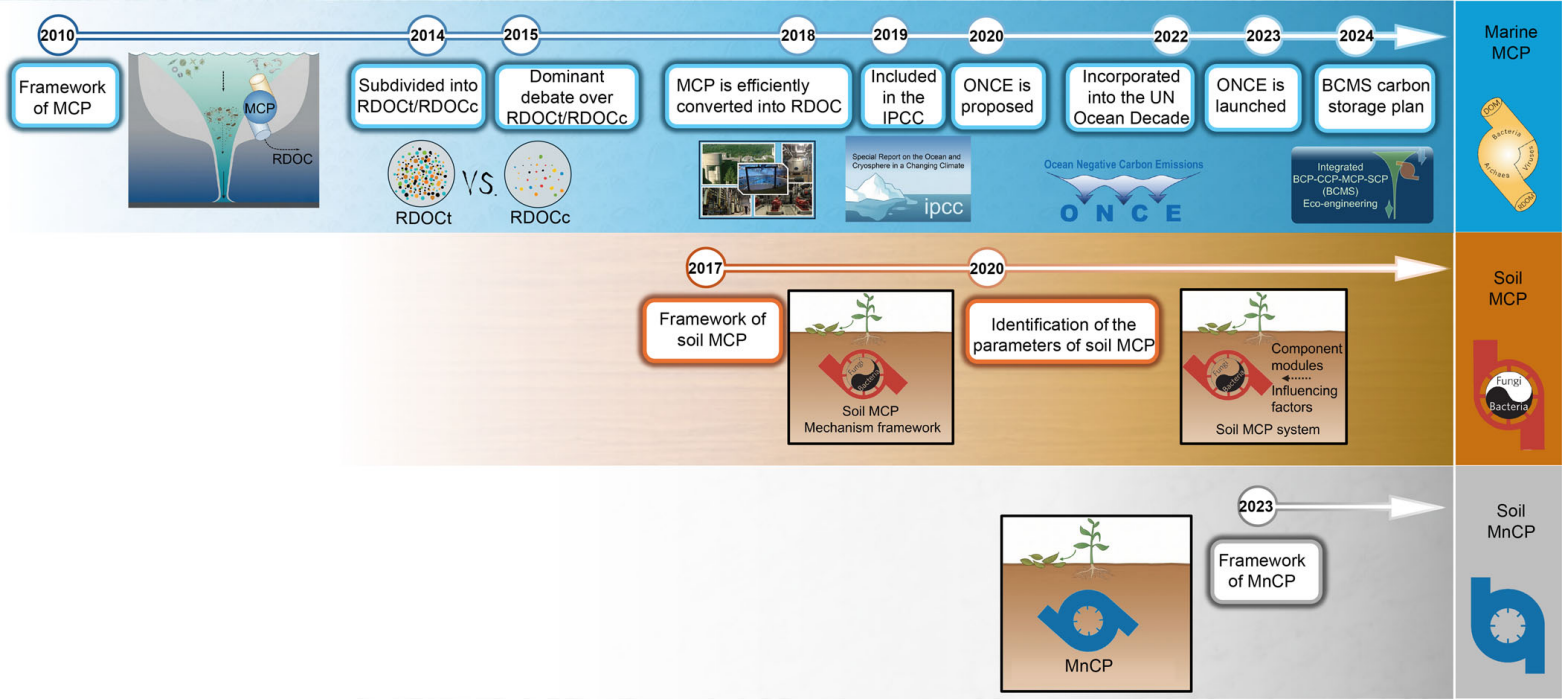
Major milestones in the evolution of the MCP concept, from its marine origin to an integrated land–sea MCP framework linking marine MCP, soil MCP, and MnCP across ecosystems. The timeline illustrates the theory's expansion from its initial marine formulation in 2010^13^ to its application in soils by 2017^16^, culminating in the recent (2023) MnCP framework for mineral‐organic systems^17^. This conceptual progression is marked by significant advances, including RDOCt/RDOCc differentiation and the establishment of MCP‐relevant soil metrics. RDOCt refers to DOC composed of inherently refractory compounds in a specific environmental context. RDOCc refers to DOC consisting of molecules at extremely low individual concentrations, below the corresponding microbial uptake thresholds. The marine MCP framework's incorporation into the IPCC assessment and global ONCE programs signals its growing centrality in global carbon sequestration efforts. BCMS, the biological carbon pump, the carbonate counter pump, the microbial carbon pump, and the solubility carbon pump; DOC, dissolved organic carbon; IPCC, Intergovernmental Panel on Climate Change; MCP, microbial carbon pump; MnCP, mineral‐associated carbon pump; ONCE, Ocean Negative Carbon Emission; RDOC, refractory dissolved organic carbon; UN Ocean Decade, United Nations Decade of Ocean Science for Sustainable Development.

The complex interplay of biological and abiotic processes across these transitional environments, particularly those mediated by microorganisms, plays a central role in regulating regional carbon cycle dynamics^9^, ^10^, ^11^. As key drivers of organic matter decomposition and formation, microorganisms exert a direct influence on global carbon balances^12^. A key mechanism is the microbial carbon pump (MCP), which facilitates the transformation of labile DOC into refractory dissolved organic carbon (RDOC), thereby enabling long‐term oceanic carbon storage^13^, ^14^, ^15^. Originally conceptualized in marine systems, the MCP concept has been extended to terrestrial environments (soil MCP) and complemented by the mineral‐associated carbon pump (MnCP) for mineral‐organic stabilization (Figure 1)^18^, ^19^. In this review, we use marine MCP to denote the original oceanic RDOC pathway and land–sea MCP framework to describe the integrated cross‐ecosystem linkage among marine MCP, soil MCP, and MnCP across the land–sea interface. Within this framework, two distinct but complementary mechanisms operate: the soil MCP, which facilitates stable soil organic carbon (SOC) formation through microbial necromass accumulation^16^, ^20^, and the MnCP, which sequesters organic carbon through physicochemical processes, including mineral adsorption, coordination, and encapsulation^17^ (Table 1). Although the MnCP has rapidly gained traction as an organizing framework, its quantitative assessment remains methodologically heterogeneous, and its coupling with the soil and marine MCP has only begun to be operationalized across the land–sea interface. Together, these mechanisms likely operate synergistically across land–sea interface zones to form an integrated carbon‐regulation system. Recognizing this pivotal role, the scientific community has emphasized that “the impact of climate change will rely heavily on responses of microorganisms, which are essential for achieving an environmentally sustainable future”^21^. It has further called for “immediate, tangible steps that harness the power of microbiology”^22^ and proposed the establishment of a global, science‐based climate task force to support the implementation of microbiome‐based technologies^23^.

**Table 1:** Convergence and divergence in carbon‐storage mechanisms among marine microbial carbon pump (MCP), soil MCP, and mineral‐associated carbon pump (MnCP).

Comparative dimension	Marine MCP	Soil MCP	MnCP
Stored carbon form	RDOC (e.g., CRAM, FDOM, and DBC)	Stabilized SOC and necromass‐origin carbon	Mineral‐associated organic carbon (MAOC)
Microbial role	Chemoautotrophic and heterotrophic metabolism and viral shunt	Fungi‐bacteria metabolism and residue formation	Biofilm‐mineral associations and microbe‐mediated organo‐mineral complexation
Mineral dependence	Low	Medium‐high	High
Stabilization pathway	Structural recalcitrance and dilution	Biogenic molecules plus mineral sorption and spatial/physical protection	Mineral sorption, polymerization, and chemical bonding
Characteristic timescale	>10^3^ years; constrained by photodegradation, hydrothermal/oxidative processes	>10–10^2^ years; modulated by soil structure and lateral loss	>10^2^–10^3^ years; influenced by burial rates and redox/oxygen conditions
Typical settings	Ocean interior and deep waters; OMZs	Surface soils, rhizosphere, tilled layers, and freeze‐thaw zones	Mineral‐rich sediments/soils
Primary controls	Substrate composition, metabolic strategies, dissolved oxygen, and water mass structure	Community composition, soil texture, moisture, and temperature	Mineralogy, pH, ionic strength/salinity, and specific surface area

**Marine MCP:** In the ocean, phytoplankton‐derived DOC is continually processed by heterotrophic bacteria, archaea, and viruses^24^, ^25^. A fraction is rapidly respired as CO_2_, whereas the remainder is incorporated into microbial biomass and transformed into metabolic byproducts. Through repeated microbial transformation, molecular selection, and dilution, a millennial‐scale RDOC reservoir accumulates, complementing the biological pump as a major pathway for dissolved carbon sequestration^13^, ^15^, ^26^. **Soil MCP:** In soils, plant‐derived organic matter is decomposed and reworked by microorganisms, generating metabolites and necromass with low bioavailability^27^, ^28^, ^29^. **MnCP:** Microbial metabolites and necromass form organo‐mineral complexes with clays and metal oxides and become physically protected within aggregates, leading to the formation of long‐term MAOC. Microbial‐derived SOC may contribute 33%–62% of total SOC^30^, ^31^. Thus, the soil MCP represents an integrated biochemical‐biophysical process, in which microbial transformation is reinforced by mineral interactions and physical occlusion. CRAM, carboxyl‐rich alicyclic molecules; DBC, dissolved black carbon; FDOM, fluorescent dissolved organic matter; OMZ, oxygen minimum zone; RDOC, refractory dissolved organic carbon; SOC, Soil organic carbon.

This review synthesizes key advances in understanding the role of microbial processes in elemental cycling and carbon cycle regulation across the Chinese land−sea interface. Furthermore, we propose an integrated study framework, grounded in the concept of multiple carbon pumps, focusing on the coexistence and synergy of MCP, soil MCP, and MnCP, which is used to study microbial carbon sequestration processes in the land−sea interface.

## MICROBIAL CARBON PROCESSING FROM RIVER TO SHELF

### River and inland freshwater systems

Freshwater microorganisms act as upstream carbon processors, rapidly remineralizing and transforming terrestrial organic matter during downstream transport. In many nutrient‐rich upper river reaches, this processing coincides with carbon (C)–nitrogen (N) decoupling, whereby nitrogen assimilation is high but denitrification remains limited^32^. This nitrogen retention, together with ongoing carbon loss, promotes phytoplankton blooms and eutrophication, shifting the DOC pool toward more bioavailable forms^33^, ^34^. Phytoplankton‐derived DOC is then rapidly converted by bacteria into CO_2_ and, within anoxic microzones, can be further converted to CH_4_ by methanogenic archaea, thereby reducing long‐term carbon storage^35^, ^36^.

Anthropogenic and climate‐related disturbances further intensify these processes. Typhoons and heavy rainfall can accelerate bacterioplankton turnover, whereas episodic inputs of organic matter and algal biomass elevate nutrient concentrations (e.g., total nitrogen, ammonium, and total phosphorus) in reservoirs, thereby stimulating microbial nitrogen removal and methanogenesis^37^, ^38^. Furthermore, microplastic pollution can elevate methanogenesis in anoxic sediments via two distinct pathways^39^, ^40^, ^41^: conventional plastics (e.g., polyethylene and polyvinyl chloride) suppress methanotrophy linked to sulfate reduction or denitrification, indirectly increasing CH_4_, whereas biodegradable plastics (e.g., polylactic acid and polybutylene adipate terephthalate) upregulate methanogenic genes such as *mcrA*, directly enhancing CH_4_ flux^40^, ^41^. Similarly, antibiotics disrupt nitrogen cycling by inhibiting denitrification and anammox and increasing the N_2_O yield under certain exposure conditions, thereby weakening sedimentary fixed‐N removal^42^. This reduced N removal can increase the potential for reactive‐N retention, which may indirectly intensify primary production and subsequent organic‐carbon loading and remineralization in nearshore waters^43^. Together, these pollutant‐induced disruptions to microbial functions create positive feedback loops that amplify greenhouse gas emissions in nearshore aquatic zones^44^.

Microbial metabolism significantly reshapes organic carbon composition during its transport through the river‐freshwater ecotone. Genomic resources like Genome Resolved Open Watersheds database (GROWdb) reveal a strong enrichment of bacterial lineages, such as *Actinomycetota*, *Pseudomonadota*, *Bacteroidota*, and *Verrucomicrobiota*, in riverine ecosystems^45^. Several of these taxa possess efficient pathways for both aerobic respiration and light‐driven energy capture, a trait clearly exemplified by the genus *Planktophila* ^46^ and members of the phylum *Actinobacteria* ^47^. These organisms use actinorhodopsin to harness light for ATP synthesis, reducing their dependence on organic carbon^47^, while *Verrucomicrobiota* encode abundant glycoside hydrolases that target complex polysaccharides, directly driving DOC deconstruction and reassembly^48^. By accelerating DOC oxidation and altering its molecular signature, these photometabolic and hydrolytic pathways thereby establish the river‐freshwater ecotone as a dynamic bioreactor for ancient terrestrial carbon. Accurate assessment of carbon‐sink efficiency across the land–sea continuum must therefore account for RDOC reactivation, microbial oxidative capacity, and pollutant‐amplified methanogenesis.

Taken together, these freshwater processes define the boundary conditions of the land–sea interface carbon budget by regulating downstream carbon export and altering substrate lability and partitioning before estuarine entry. How these boundary conditions propagate through estuarine and coastal transformation pathways is discussed in the following sections.

### Mangroves and tidal flats

Coastal wetlands and tidal flats are key land−sea interfaces that receive terrestrial inputs and regulate organic matter cycling, ranking among the most powerful yet sensitive carbon‐sequestration systems^49^, ^50^. Their role in moderating land−sea carbon fluxes is now understood to be significantly more complex than previously assumed^51^, ^52^. In mangroves in particular, sediment–tidal exchange and microbial activity drive intense biogeochemical processing, generating a multi‐interface carbon sink sustained by tightly coupled processes^53^, ^54^.

Multi‐omics and co‐occurrence network analyses indicate that sulfate‐reducing taxa constitute a dominant functional guild in mangrove sediments, and >70% of the identified keystone and hub taxa in co‐occurrence networks were sulfate‐reducing prokaryotes^55^, while denitrification and dissimilatory nitrate reduction to ammonium display strong vertical stratification. This organization forms functional modules centered on sulfur and nitrogen reduction, which can regulate greenhouse‐gas fluxes by structuring sulfide‐linked, often incomplete denitrification with elevated N_2_O production potential and by shifting depth‐dependent carbon mineralization and methane cycling under low‐oxygen conditions^56^, ^57^, ^58^. Mangroves often function as net CO_2_ sinks and CH_4_ sources, with CH_4_ emissions increasing under anthropogenic disturbances (e.g., aquaculture effluent) that introduce labile organic carbon and stimulate methanogenic consortia^58^, ^59^, ^60^. Along depth gradients, sulfate reducers, denitrifiers, and methane‐cycling microbes efficiently couple carbon, nitrogen, and sulfur cycling. This metabolic network reassembles terrestrial DOC into more aromatic, sulfur‐ and nitrogen‐enriched recalcitrant molecules, while simultaneously regulating greenhouse gas production and consumption^61^, ^62^. The concept of microbial active functional modules has been proposed to describe these interactions, distinguishing surface layers dominated by nitrate reduction from deeper layers driven by complex organic‐carbon degradation. Cross‐module and environment‐module exchanges are considered crucial for maintaining stable carbon storage^63^.

Vegetation shifts reshape sediment biogeochemistry by altering nutrient supply and redox microenvironments, thereby reorganizing coupled nitrogen, sulfur, and methane processes. *Spartina alterniflora* can significantly increase summer NH_4_^+^ and NO_3_^−^ concentrations^64^, thereby modifying substrate availability for nitrogen transformations. Under these conditions, pH and acid‐volatile sulfide jointly regulate sulfur oxidation and denitrification, and sulfide can act as a potential driver of N_2_O production in surface sediments^58^. With depth, elevated abundances of *dsrAB* and *mcrA* indicate strengthened sulfate reduction and methanogenesis under more persistent hypoxia^58^. Similarly, although the introduction of *Sonneratia apetala* increases canopy cover, it can restructure methanogen communities and enhance CH_4_ emissions, and methanogen assemblages in deep hypoxic sediments may retain nitrogen‐fixation potential^60^, ^65^.

Tidal flats (intertidal mud and sand flats) are now recognized as major blue‐carbon systems, with a global extent exceeding 120,000 km^2^ and an average carbon burial rate comparable to mangroves and salt marshes^66^. Seasonal variations in temperature and hydrology drive temporal shifts in DOC sources and properties, as reflected in seasonal differences in ultra violet (UV) absorbance, aromaticity, fluorescence indices, and molecular weight^67^, ^68^. Sediment microbiomes play a key role in DOC degradation and reassembly, exhibiting latitudinal declines in both DOC molecular and microbial diversity^69^, ^70^. Meanwhile, viruses carrying auxiliary metabolic genes can reprogram host metabolism and participate directly in elemental cycling^69^, ^70^. At the molecular level, microbial processing promotes the chemical and structural complexity of DOC, generating low‐bioavailability, recalcitrant fractions that contribute to RDOC formation and long‐term carbon storage, potentially including enhanced sedimentary sequestration. Certain viral auxiliary metabolic genes may further stimulate host carbon fixation and organic synthesis, increasing burial efficiency^71^. Spatial gradients in salinity, oxygen, and root activity from subtidal to supratidal zones further drive functional turnover in microbial communities. The longstanding underestimation of productive biofilms and chemoautotrophic pathways likely has led to systematic underestimation of regional carbon‐sink strength and greater uncertainty in the source apportionment of stored carbon^68^, ^72^.

### Estuaries and the continental shelf

The estuary–continental shelf nexus is a critical transition zone characterized by high productivity, substantial carbon fluxes, and intense sedimentary processing^73^. At seasonal timescales, reduced nutrient limitation and efficient processing of photosynthate modulate CO_2_ uptake^74^. On annual timescales, the net carbon sink of the eastern Chinese shelf is maintained primarily by circulation‐driven cross‐shelf carbon export^75^. Long‐term observations indicate that POC fluxes across the Pearl River Estuary–East China Sea continuum are governed by terrestrial inputs, phytoplankton blooms, and sediment resuspension, generating pronounced seasonal‐to‐interannual variability and strong vertical differentiation^76^, ^77^. Crucially, the transformation of this organic matter is mediated by distinct microbial functional groups that track these dynamic physical conditions. In productive surface waters and during resuspension events, heterotrophic bacteria, particularly flavobacterial lineages and *Rhodobacteraceae*, are rapidly enriched^78^, ^79^, ^80^, ^81^. These taxa drive rapid turnover of labile substrates, a process associated with DOC reactivation and consistent with partial RDOC production^79^, ^82^.

In hypoxic to anoxic bottom waters, microbial respiration shifts from aerobic pathways to anaerobic metabolism, while reoxidation of reduced intermediates can fuel chemoautotrophic dark carbon fixation^83^, ^84^. Chemoautotrophic dark carbon fixation can locally approach photosynthetic uptake in anthropogenically influenced estuaries. In the Yangtze Estuary and adjacent coastal waters, dark carbon fixation accounted for 15.4%–97.7% of integrated daily total carbon fixation, and microorganisms bearing the form IA and IC ribulose‐1,5‐bisphosphate carboxylase/oxygenase large subunit genes (*cbbL*‐IA & IC) were identified as potential key contributors^85^, ^86^, highlighting an often underappreciated contribution to regional carbon inventories.

Nitrogen‐loss pathways are also microbially mediated. In oxygen‐depleted sediment transition zones, anammox can contribute substantially to fixed‐nitrogen removal and may account for up to ~50% of total nitrogen loss in East China Sea shelf sediments^86^. Meanwhile, particle‐associated communities form more complex and stable networks than free‐living assemblages^87^, ^88^, and can efficiently process resuspended organic matter, thereby modulating MCP efficiency in shelf‐sea environments^89^, ^90^.

**Figure 2:**
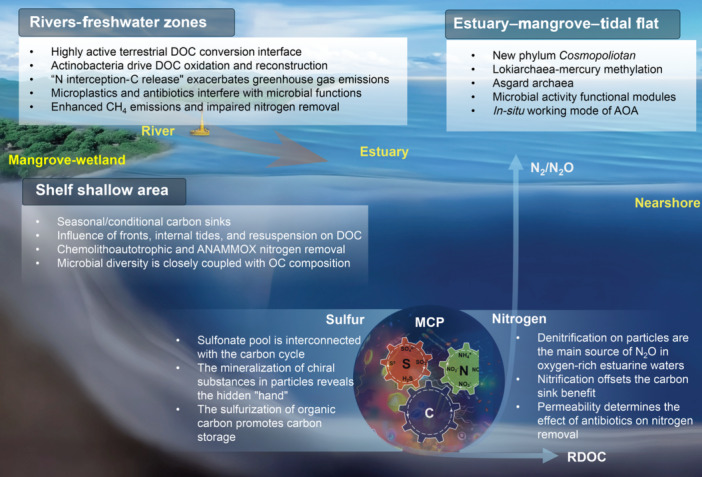
Key recent advances in biogeochemical dynamics and microbial processes along the mangrove–estuary–nearshore continuum, and their implications for ocean carbon cycling. The schematic highlights major research foci related to the MCP across the land–sea interface, tracing the continuum from rivers and freshwater systems through estuaries, mangroves, and tidal flats to the continental shelf. Key processes include DOC reactivation, transitions between net carbon sink and source states, and coupling among the carbon, nitrogen, and sulfur cycles. ANAMMOX, anaerobic ammonium oxidation; AOA, ammonia‐oxidizing archaea; OC, organic carbon.

In summary, observed sink‐source reversals of air‐sea CO_2_ fluxes across Chinese shelf seas^74^, ^91^ underscore the need to understand the underlying microbial and biogeochemical mechanisms. Key knowledge gaps include the controls on MCP efficiency, DOC molecular transformation pathways, and benthic–pelagic interactions involving hypoxia, nitrogen loss, and sulfur oxidation. Such mechanistic understanding is essential for accurate sink quantification, clarification of sequestration pathways (Figure 2), and can further inform the development of effective policy interventions.

## MECHANISMS OF ORGANIC CARBON TRANSFORMATION AND SEQUESTRATION AT THE LAND−SEA INTERFACE

**Figure 3:**
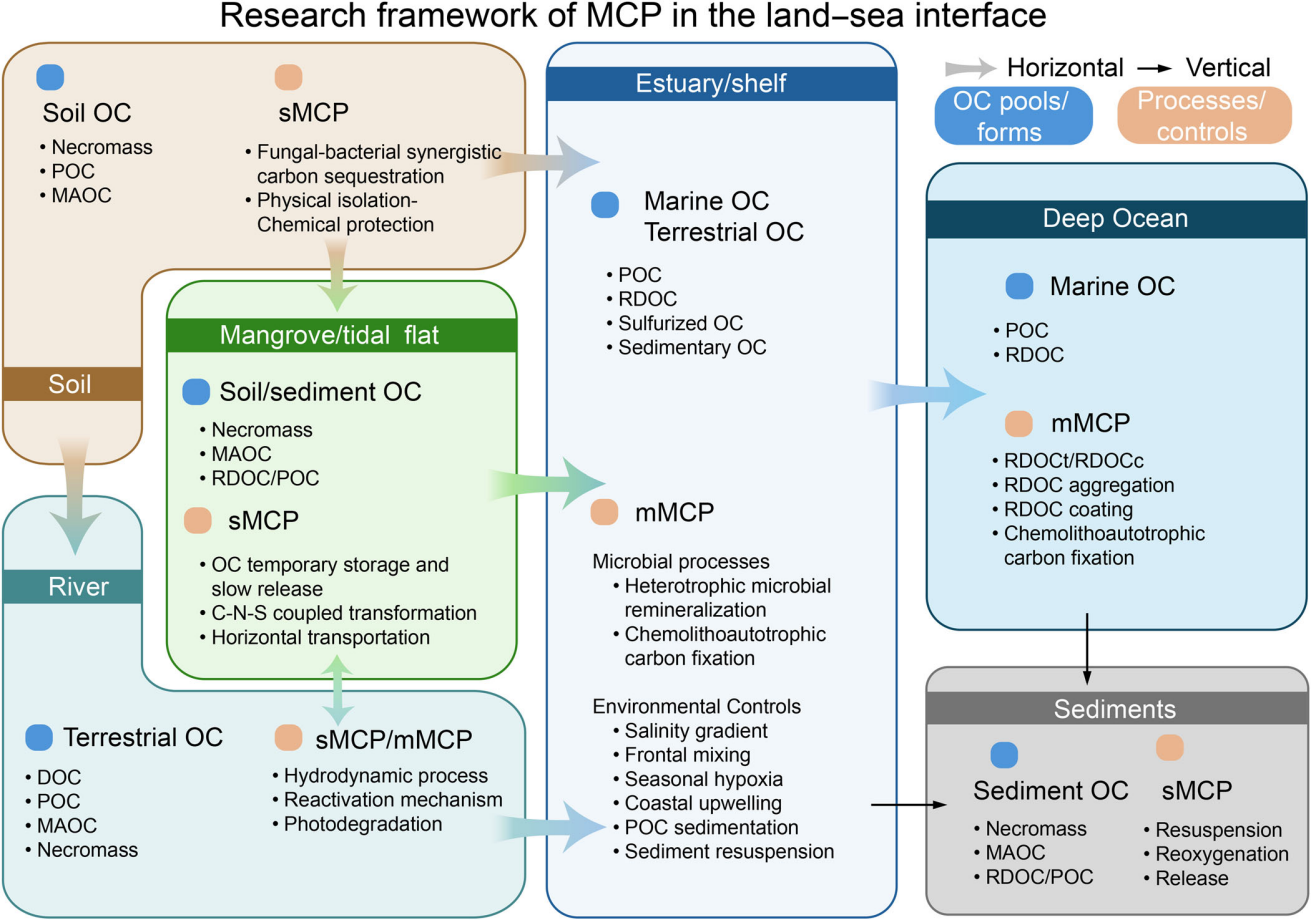
Carbon transfer across the land−sea interface. Carbon forms include microbial necromass, MAOC, and RDOC. Key processes illustrate the sequential microbial transformation, stabilization, and redistribution of carbon across ecosystem compartments. MAOC, mineral‐associated organic carbon; mMCP, marine MCP; POC, particulate organic carbon; sMCP, soil MCP.

Microbial processes continuously redistribute and transform organic carbon across successive land−sea interface compartments, with key thresholds—such as hypoxia, sedimentation intensity, and salinity gradients—modulating molecular composition and stability (Figure 3).

In upland systems, the soil MCP regulates plant‐fungi‐bacteria interactions and promotes initial organic carbon stabilization. Microbial communities in the rhizosphere and surface soils transform plant‐derived polysaccharides and lignin into metabolic intermediates and necromass^92^, ^93^, which associate with clays and metal oxides to form organo‐mineral complexes. These are protected within aggregates, contributing to mineral‐associated organic carbon (MAOC) formation^31^, ^94^. Field studies show that fungi with high carbon‐use efficiency can enhance SOC stocks while reducing N_2_O emissions from denitrification^95^, ^96^, ^97^. At deeper soil layers (20–50 cm), often under mild acidity and low oxygen, carbon stabilization is further strengthened through interactions with Fe/Al oxides and clays^98^, ^99^, ^100^, ^101^. Globally, MAOC stocks and turnover constrain downstream carbon inputs, estimated at ~7.6 Pg C yr^−1^ ^102^.

Rivers are now recognized not as passive conduits but as active biogeochemical reactors that significantly alter terrestrial carbon during transport^103^, ^104^, ^105^. Globally, rivers export ~1.02 ± 0.22 Pg C yr^−1^, including 0.30 ± 0.14 Pg C yr^−1^ as DOC and ~0.18 ± 0.04 Pg C yr^−1^ as POC^106^. This paradigm shift is quantified by the fate of terrestrial carbon inputs: about 40% is returned to the atmosphere via inland‐water evasion, 12% buried, and only 48% reaches the ocean, necessitating the inclusion of freshwater carbon reservoirs in terrestrial budgets^103^. Atmospheric evasion is dominated by CO_2_, but it also includes CH_4_ that can be especially important in anoxic inland waters; rivers and streams alone emit about 27.9 Tg CH_4_ yr^−1^ ^107^, ^108^. The reactivity of specific carbon fractions underscores this role. For example, global rivers deliver ~0.58–1.2 Tg C yr^−1^ of dissolved lignin‐like compounds to coasts^109^, yet this seemingly recalcitrant carbon is rapidly degraded in transit—photodegradation can remove up to 72% within 10 days, drastically reducing its long‐term sequestration potential^110^, ^111^. Furthermore, soil‐derived DOC mobilization is highly dynamic, driven by storms, freeze‐thaw cycles, and land‐use change, with soil‐riverine residence time of months to years in mid‐high latitudes^112^, ^113^. Rainfall, groundwater discharge, and surface runoff transport DOC, POC, and associated microbes into rivers, where they undergo dilution and microbial reprocessing^114^, ^115^. Although small and medium watersheds cover only ~3% of global land area, they may contribute 17%–35% of riverine organic carbon^109^, ^116^, ^117^. Groundwater provides ~5%–20% of riverine DOC, often accompanied by reduced species such as Fe^2+^/Mn^2+^ ^118^, ^119^, ^120^, ^121^. Photoheterotrophic and aerobic bacteria (e.g., *Actinobacteria* and *Bacteroidota*) rapidly transform lignin‐rich terrestrial DOC into carboxyl‐rich alicyclic molecules (CRAM), a process often coupled with nitrate assimilation^122^, ^123^. In estuaries, salt‐induced flocculation settles ~10%–30% of aromatic DOC with fine particles^124^, ^125^. Beyond physicochemical partitioning, salinity acts as a strong environmental filter that reorganizes estuarine microbiomes and their carbon‐processing traits, thereby altering the accessibility and microbial transformation pathways of terrigenous DOC across the freshwater−seawater transition^126^, ^127^, ^128^.

Mangroves serve as a critical transition in the land−sea continuum, linking upstream soil mineral‐necromass complexes with estuarine salinity gradients. Their carbon sequestration capacity per unit area exceeds that of terrestrial forests^129^. High productivity and thick sediments allow short‐term retention and reprocessing of allochthonous and autochthonous organic matter. Globally, it is estimated that mangroves export ~24 ± 21 Tg C yr^−1^ as DOC and ~22 ± 27 Tg C yr^−1^ as POC, along with significant substantial exports of dissolved inorganic carbon outflow^51^, ^130^, ^131^, ^132^, ^133^, ^134^. Estuarine mangroves store more SOC (282 Mg C ha^−1^) than oceanic mangroves (250 Mg C ha^−1^), indicating effective interception of terrestrial carbon^54^. Net ecosystem production averages ~794 g C m^−2^ yr^−1^, with ~5% buried long‐term^135^. Microbial communities mineralize part of the organic matter and convert another fraction into macromolecules or necromass, which—when protected by physicochemical mechanisms—form stable carbon pools^136^, ^137^. Mangrove soils contain high microbial necromass carbon (10.9 mg C g^−1^), far exceeding salt marshes (~1.9 mg C g^−1^), with fungal residues dominating the recalcitrant fraction^138^.

On the continental shelf, the marine MCP governs the fate of organic carbon. Phytoplankton‐microbe consortia dominate surface production, while hypoxic bottom waters promote dark carbon fixation by chemoautotrophs^85^, ^139^. Heterotrophic microbes enhance the refractory‐to‐labile DOC ratio and produce humic‐like fluorescent DOC and CRAM via enzymes such as polysaccharide lyases and phenol oxidases^140^. Functional redundancy and species turnover sustain these processes. The real‐time response of key metabolic modules—dark carbon fixation, sulfur oxidation, and aromatic cleavage—to salinity, oxygen, or disturbance determines the rate of refractory carbon formation^85^, ^141^. Viral and plasmid‐borne auxiliary metabolic genes may further accelerate RDOC production^142^, ^143^. Approximately 20% of this carbon is transported to depths >1000 m via sinking and currents, enabling millennial‐scale sequestration^144^. Recent evidence supports the RDOCt concept (intrinsic RDOC generated under specific biotic and abiotic environmental conditions), thereby challenging the Dilution Hypothesis and suggesting a dynamic balance between RDOC formation and substrate turnover^145^, ^146^.

## AN INTEGRATED CARBON CYCLE STUDY FRAMEWORK FOR THE LAND−SEA INTERFACE

### MCP and MnCP

Land–sea interface dynamics—including salinity gradients, hypoxia, sediment resuspension, and terrestrial inputs—reshape carbon pools by mobilizing organic fractions, accelerating remineralization, and promoting RDOC formation. Microbial activity at mineral surfaces further regulates carbon fate, determining whether organic carbon is stabilized or returned to the atmosphere as CO_2_. As the MCP framework has expanded, single‐environment perspectives have become insufficient to explain the microbially mediated fate of organic carbon. Together, the MCP and MnCP provide a unifying framework that links microbial and mineral transformation pathways, including RDOC formation, necromass production, and MAOC accumulation, to carbon stabilization across ecosystems. Both marine and soil MCPs are grounded in the principle that microbial activity governs carbon persistence. In marine systems, stabilization is dominated by molecular recalcitrance, whereas in soils it is dominated by mineral association, necromass accumulation, and physical protection.

The synergistic interplay between the MCP and MnCP can be conceptualized as two primary modes: the serial pathway and the parallel pathway^147^, ^148^. The serial pathway is often interpreted as a sequestration‐oriented mechanism, in which the MCP generates relatively inert organic carbon, and the MnCP subsequently stabilizes this material through mineral association; in this process, microbial residues enriched in polar functional groups exhibit high affinity for mineral surfaces, thereby promoting stronger stabilization via ligand exchange and cation bridging^149^, ^150^. By contrast, the parallel pathway involves direct mineral shielding of relatively unprocessed organic matter; although this can contribute to carbon storage, the resulting complexes may not necessarily achieve stability comparable to microbially processed MAOC formed through the serial pathway^148^.

At the land−sea interface, carbon‐stabilization pathways co‐occur and become sequentially coupled. In soils, microbial processing converts plant inputs into microbial necromass that is preferentially stabilized as mineral‐associated organic carbon on reactive mineral phases^93^. Across the freshwater–seawater transition, salinity shifts and redox‐driven iron cycling can remobilize part of mineral‐bound organic matter into the dissolved pool, increasing the availability of terrigenous substrates for coastal microbial processing^151^. Microbial necromass pools can be remobilized during land‐to‐coast transport, and then either preserved through rapid burial under high‐sedimentation, hypoxic conditions, or further microbially remodeled into RDOC or remineralized to CO_2_ ^26^, ^98^, ^152^. Once mobilized, these substrates can be rapidly remineralized or further transformed through the marine MCP into more persistent dissolved products that contribute to RDOC formation and modulate nearshore burial efficiency^110^, ^147^. Accordingly, carbon fate is jointly shaped by soil MCP/MnCP preprocessing and marine MCP remodeling, highlighting a sequential control system operating across the continuum.

Integrating land−sea MCP framework into carbon budget models for the land−sea interface requires a clearer understanding of the fate of terrigenous organic carbon during cross‐boundary transport—a central challenge in coupling carbon pumps across environmental media. This effort must reconcile three fundamental differences between soils and marine systems—substrate type, dominant microbial communities, and physicochemical conditions—while recognizing their shared principle: the microbial conversion of organic carbon from labile to refractory forms^13^, ^16^.

Several mechanistic parallels underline microbial carbon transformation and stabilization across these systems. First, complexifying biosynthesis—microbial production of extracellular polymeric substances and metabolites increases molecular complexity and resistance to degradation^153^. Second, assimilatory transformation—incorporation of exogenous organic carbon into microbial biomass, including both living cells and necromass, contributes to stabilized carbon pools^149^, ^154^. Third, the use of diverse electron acceptors—under hypoxic or anoxic conditions, microorganisms use electron acceptors such as nitrate and sulfate to drive decomposition and chemoautotrophy, often generating reduced intermediates with enhanced stability^155^, ^156^, ^157^. Fourth, physical protection—physicochemical mechanisms limit microbial access to organic matter; in soils, minerals and aggregates protect MAOC, whereas in aquatic systems, colloids and fine particles promote the adsorption, aggregation, and sedimentation of organic matter^17^, ^26^.

### Core quantification metrics

To operationalize the land−sea MCP framework in models and observations, a practical next step is parameterization. Within the land−sea MCP framework, carbon use efficiency (CUE) and bacterial growth efficiency (BGE)—commonly applied in soil and aquatic systems, respectively—quantify the partitioning of assimilated organic carbon between biomass production and respiration, thereby governing microbial metabolic routing and carbon fate, despite differences in their measurement protocols^158^, ^159^. In marine systems, the MCP has motivated quantitative work on microbially produced RDOC and bacterial detritus as climate‐relevant storage forms^13^, ^160^. In this context, BGE is most commonly quantified experimentally by estimating bacterial production from ^3^H‐leucine or ^3^H‐thymidine incorporation, while respiration is inferred from oxygen consumption or dissolved inorganic carbon production under dark incubations^159^, ^161^, ^162^.

In terrestrial systems, CUE is typically estimated using ^13^C‐labeled substrates by partitioning tracer carbon between microbial biomass growth (e.g., biomass quantified via chloroform fumigation‐extraction, phospholipid fatty acid analysis, or DNA‐based biomass metrics) and losses as ^13^CO_2_ ^163^; complementary approaches include growth estimation based on H_2_^18^O incorporation and, under specific assumptions, model‐based inversion to derive an effective CUE^164^. A pivotal conceptual advance in this field has been the recognition that microbial residues constitute major precursors of MAOC^31^, ^99^. Consequently, the quantification of microbial residues has evolved along three main lines: (i) bulk microbial biomass proxies (e.g., chloroform fumigation‐extraction and phospholipid fatty acid [PLFA] analysis)^165^, ^166^; (ii) biomarker‐based residue accounting—most notably using amino sugars and muramic acid to infer bacterial versus fungal residue contributions^167^; (iii) isotope‐enabled tracking of the turnover and fate of microbially derived carbon entering mineral‐associated pools^168^, ^169^.

Parallel advancements have occurred in aquatic systems. d‐amino acids and muramic acid have been used as bacterial biomarkers to estimate the contributions of living and detrital bacteria to particulate and DOC reservoirs^170^, ^171^. These biomarkers complement approaches that operationalize RDOC via bulk DOC and Δ^14^C inventories, as well as molecular‐level characterizations (e.g., Fourier Transform Ion Cyclotron Resonance Mass Spectrometry)^79^. Overall, these methodological advances make it possible to explicitly link BGE‐ and CUE‐mediated carbon partitioning to measurable microbial‐derived end products, rather than inferring persistence indirectly from bulk carbon pools.

CUE and BGE thus quantify the balance between CO_2_ released through respiration and carbon incorporated into microbial biomass—the efficiency of synthesis versus respiration. Accordingly, these efficiencies constrain how much organic carbon is routed toward microbial biomass and necromass, a key precursor to mineral‐associated and stable SOC in the soil MCP. By contrast, there remains a lack of explicit quantification of the extracellular by‐product stream that can be transformed into RDOC via the MCP pathway.

CUE is typically defined as the fraction of assimilated carbon allocated to microbial biomass:

$$\text{CUE}\;=\;\frac{C_{\text{growth}}}{C_{\text{growth}}+C_{\text{resp}}}$$

Where *C*_growth_ represents carbon incorporated into biomass, and *C*_resp_ denotes carbon lost through respiration^172^.

BGE, widely used in aquatic microbiology, is analogously defined as the ratio of bacterial production (BP) to the sum of production and bacterial respiration (BR)^159^, ^173^

$$\text{BGE}\;=\;\frac{\text{BP}}{\text{BP}+\text{BR}}$$

In the absence of other carbon losses, BGE is mathematically equivalent to CUE, and the terms are sometimes used interchangeably in soil literature^174^. However, microbes often divert a substantial share of assimilated carbon to extracellular by‐products such as secretions, enzymes, mucus, and polysaccharides, which are not captured as biomass. When these by‐products are significant, CUE exceeds BGE by an amount proportional to the excreted carbon fraction:

$$\text{CUE}\;=\;\text{BGE}+\frac{C_{\text{by}‐\text{prod}}}{C_{\text{intake}}}$$

where *C*_by‐prod_ is the excreted organic‐carbon by‐product and *C*_intake_ is total carbon assimilated^175^.

In well‐aerated soils, extracellular carbon secretion is generally minimal and often disregarded, allowing CUE to be approximated as BGE^174^, ^176^. In aquatic systems, however, excretion can be substantial; for example, marine heterotrophic bacteria may release 10%–30% of assimilated carbon as DOC. Consequently, BGE based solely on biomass and respiration underestimates total carbon utilization^170^, ^177^, ^178^. Correcting for DOC excretion raises the effective CUE relative to traditional BGE, effectively augmenting BGE with a by‐product term^174^.

Taken together, integrating the two efficiency formulations through the by‐product term provides a unified parameterization of microbial carbon routing across the land−sea interface. Within this framework, BGE constrains the biomass branch that ultimately contributes to necromass formation and mineral association, whereas the difference between CUE and BGE represents the by‐product branch that can subsequently be transformed into RDOC through the marine MCP^160^, ^174^. Because mineral‐stabilized necromass and RDOC represent the two dominant forms of persistent organic carbon in these environments, this integrated identity directly links microbial metabolism to the two sequestration outcomes most relevant to the land−sea carbon balance.

When applying CUE and BGE within the land−sea MCP framework, system‐specific contexts must still be incorporated. For example, in coastal wetlands, lateral carbon transport and hydrodynamic disturbance must be explicitly represented to accurately capture the effects of variation in microbial efficiency on carbon pools. Equally important are alignment of observational and model timescales, differentiation of microbial communities with functional‐group weighting, and incorporation of key physicochemical controls, including temperature, pH, and dissolved oxygen, among others. In sunlit estuarine and nearshore waters, photochemical mineralization and photo‐oxidative transformation occur alongside microbial processing, directly converting a fraction of DOC to CO_2_ and altering DOC lability before microbial uptake^179^, ^180^. Within this framework, these abiotic pathways can be treated as upstream modifiers that reshape the effective substrate pool and, consequently, the apparent BGE or CUE.

Although CUE and BGE provide mature metrics for the biological dimension of carbon cycling, the MnCP still lacks comparably standardized experimental approaches for quantifying its efficiency. At present, MnCP assessment relies primarily on static pool‐fractionation approaches and adsorption isotherms, which quantify storage capacity rather than process rates^181^, ^182^. This methodological gap arises because the MnCP encompasses complex, scale‐dependent mechanisms, ranging from multilayer sorption (the “onion‐skin” model) to co‐precipitation^183^. Future work should therefore move beyond static inventories toward dynamic metrics that couple microbial turnover rates with mineral saturation potential, thereby enabling fuller integration of the MnCP into the land−sea MCP framework.

## PROSPECTS FOR FUTURE RESEARCH

Substantial advances over the past decade have elucidated the critical role of microbial processes in regulating carbon cycling and greenhouse gas fluxes along the land−sea interface. The development of the land−sea MCP framework now enables systematic tracking of carbon transformation from terrestrial systems to the ocean interior. Integrated applications of metagenomics, Fourier Transform Ion Cyclotron Resonance Mass Spectrometry, and multi‐isotope techniques have identified key biogeochemical thresholds controlling carbon fate across ecosystem boundaries.

This synthesis emphasizes three critical research priorities: (i) developing unified model architectures that represent microbial carbon transformation across the land−sea interface; (ii) implementing coordinated observing systems with improved temporal resolution to capture threshold responses across ecosystems; (iii) advancing data–model integration to explicitly represent RDOC formation and mineral‐stabilization mechanisms.

Addressing these priorities will help establish verifiable accounting frameworks for microbially mediated carbon sequestration, thereby supporting its integration into climate policy and blue‐carbon assessment. This research trajectory would provide a stronger scientific foundation for nature‐based climate solutions.

**Figure 4:**
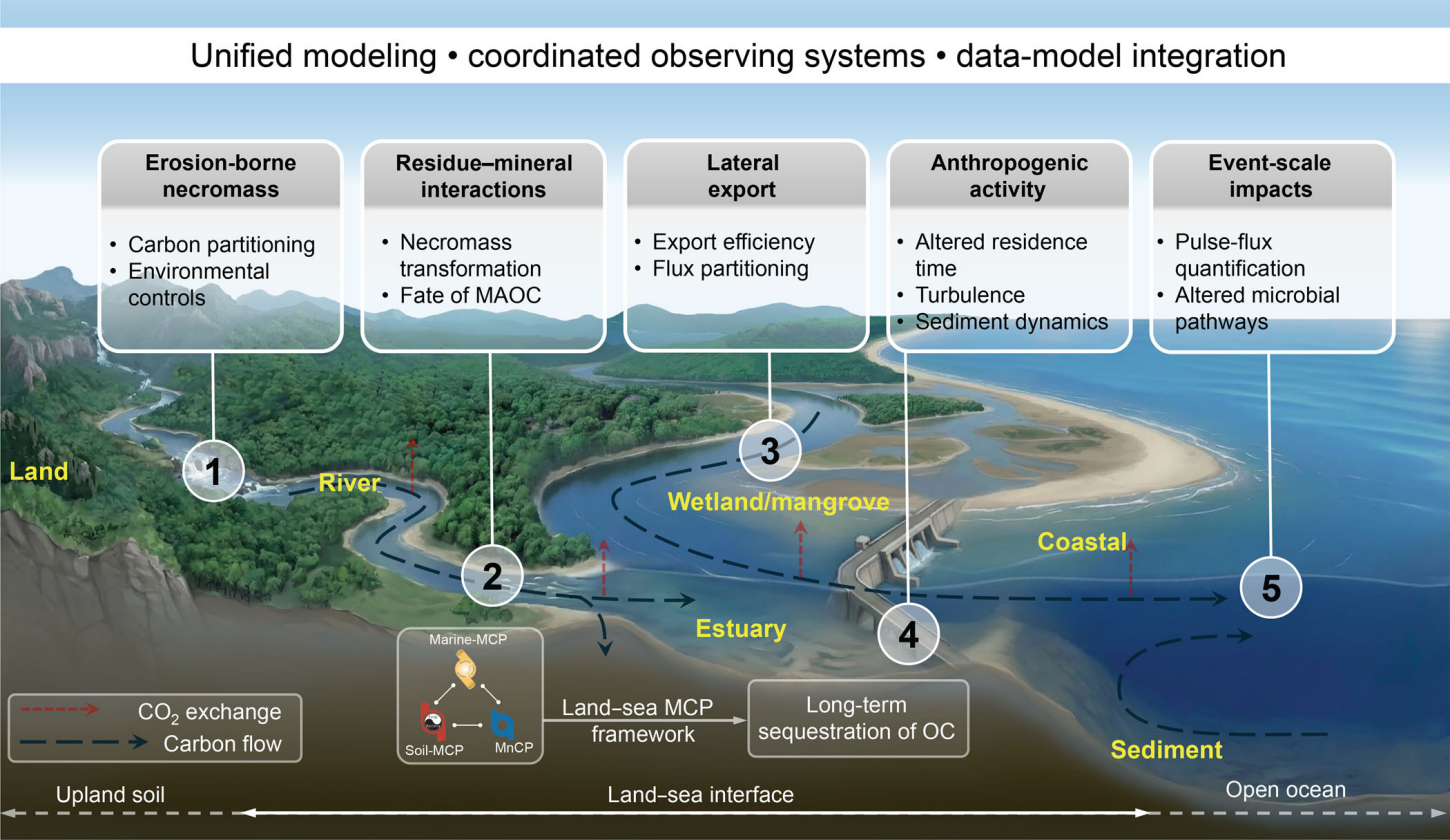
Conceptual schematic of five control themes regulating organic carbon transformation, transport, and sequestration across the river–estuary–shelf continuum. The numbered labels indicate five priority themes for advancing the land–sea MCP framework: 1, partitioning of erosion‐borne microbial necromass among mineral‐associated storage, dissolved/colloidal export, and remineralization; 2, residue–mineral interactions across the freshwater–seawater transition; 3, process efficiency and lateral carbon export in wetlands and mangroves; 4, anthropogenic reconfiguration of coastal hydrodynamics and sediment dynamics; and 5, event‐scale impacts and pulse fluxes driven by storms, resuspension, and other episodic disturbances. Black dashed arrows denote organic carbon flow along the land–river–estuary–coastal ocean continuum, whereas red dashed arrows indicate CO_2_ exchange. The lower schematic links soil MCP, marine MCP, and mineral‐associated stabilization within the land–sea MCP framework and indicates their contribution to long‐term organic carbon sequestration.

To further advance the land−sea MCP framework, future work should more tightly connect microbial reprocessing of organic matter to downstream stabilization and cross‐interface transport along the river–estuary–shelf continuum. Clarifying where, when, and under which boundary conditions microbial transformation shifts carbon toward persistent rather than rapidly recycled forms will be essential for improving conceptual coherence and predictive accounting across interfaces (Figure 4).

1. Partitioning of erosion‐borne microbial residues. What fraction of erosion‐ and runoff‐delivered microbial residues is stabilized into mineral‐associated pools versus exported in dissolved/colloidal forms versus rapidly remineralized, and which factors (e.g., mineral reactivity, transport time, and redox exposure) govern this partitioning across day‐to‐year timescales?
2. Residue‐mineral interactions across the fresh‐saline transition. How do salinity‐ and redox‐driven shifts in Fe/Al (oxyhydr)oxides and clay mineral surfaces regulate the binding mode and reversibility of microbial residues during river‐to‐estuary transport, and can we constrain sorption/desorption and transformation kinetics that predict persistence as MAOC versus remobilization to DOC?
3. Process efficiency and lateral export in mangroves. What is the net efficiency with which mangrove sediments convert incoming organic matter into RDOC and mineral‐associated pools relative to CO_2_/CH_4_ losses, and what controls the partitioning between vertical retention and lateral export across the soil–water interface?
4. Anthropogenic hydrodynamic reconfiguration of coastal MCP. How do human‐driven changes in residence time, turbulence, and sediment dynamics (e.g., reclamation, breakwaters, and channelization) shift MCP throughput (DOC degradation vs. restructuring toward RDOC), alter particle‐DOC exchange, and re‐route the magnitude and direction of lateral carbon export on continental shelves?
5. Event‐scale impacts and pulse fluxes. To what extent do episodic events (e.g., resuspension and storms) reset microbial pathways and redox microenvironments, and how do these disruptions generate pulse fluxes of DOC/POC and greenhouse gases?
